# Seasonality and social factors, but not noise pollution, influence the song characteristics of two leaf warbler species

**DOI:** 10.1371/journal.pone.0257074

**Published:** 2021-09-02

**Authors:** Krzysztof Deoniziak, Tomasz S. Osiejuk

**Affiliations:** 1 Department of Behavioural Ecology, Institute of Environmental Sciences, Faculty of Biology, Adam Mickiewicz University, Poznań, Poland; 2 Laboratory of Insect Evolutionary Biology and Ecology, Faculty of Biology, University of Bialystok, Białystok, Poland; Wildlife Conservation Society Canada, CANADA

## Abstract

Changes in the acoustic signalling of animals occupying urban ecosystems is often associated with the masking effects of noise pollution, but the way in which they respond to noise pollution is not straightforward. An increasing number of studies indicate that responses can be case specific, and some species have been found to respond differently to high levels of natural versus anthropogenic noise, as well as different levels of the latter. While the perception of noise between species may vary with its source, amplitude and temporal features, some species may possess broader environmental tolerance to noise pollution, as they use higher frequency vocalizations that are less masked by low-frequency urban noise. In this study, we explored the song variation of two closely related leaf warblers, the Common Chiffchaff *Phylloscopus collybita* and the Willow Warbler *Phylloscopus trochilus*, inhabiting urban green spaces and nonurban forests. The main goal of our study was to evaluate the impact of moderate levels of noise pollution on the songs of species which use higher frequency vocalizations and large frequency bandwidth. Previous studies found that the Common Chiffchaff modified their song in response to intense noise pollution, while no such data is available for the Willow Warbler. However, the majority of urban green spaces, which serve as wildlife hot spots in urban environments are usually polluted with moderate noise levels, which may not mask the acoustic signals of species that communicate with higher frequency. We analysed the spectral and temporal song parameters of both warblers and described the ambient noise present in males’ territories. Additionally, we looked at the social and seasonal aspects of bird song, since there is more than just noise in urban ecosystems which may affect acoustic communication. We found no evidence for noise-related bird song divergence in either species, however, we showed that social factors, time of day and season influence certain Common Chiffchaff and Willow Warbler song characteristics. Lack of noise-related bird song divergence may be due to the relatively low variation in its amplitude or other noise features present within the song frequency range of the studied species. Similar results have previously been shown for a few songbird species inhabiting urban ecosystems. Although in many cases such results remain in the shadow of the positive ones, they all contribute to a better understanding of animal communication in urban ecosystems.

## Introduction

Urbanization results in various ecological and environmental issues, which may lead to local species extinctions [1–4] and changes in physiological and behavioural traits [5–9], as well as the disconnection of human society from nature [10]. One of the major consequences of urban development is noise pollution, which is generated from our everyday activities such as transportation, industry and recreation, and can vary significantly over time and space [11, 12]. The impact of noise pollution is multifaceted and detrimental to humans (reviewed in [13]) and wildlife (reviewed in [14, 15]). Noise pollution notably impairs communication by masking acoustic signals [16], which hinders the ability of aquatic and terrestrial organisms to detect and decode messages [14], causing consequences for individuals and on a population level (e.g. [17–19]).

Bird song is one of the most fascinating examples of animal communication, utilised by songbirds for mate attraction and territorial defence [20]. This complex form of communication is shaped by various factors such as sexual selection [21], population structure [22], habitat complexity [23], and habitat quality [24]. However, noise pollution creates evolutionary novel acoustic environments, which disrupt avian vocal communication [25], eventually leading to avian species decline in urban ecosystems [4]. Previous studies have found that birds living in human-altered environments were shown to modify song spectral characteristics [26], song duration and song rate [27], song complexity [28], vocal output [29] and timing of singing [30].

Observed variation in song characteristics between birds inhabiting urban and nonurban populations is often associated with the masking effect of anthropogenic noise (e.g., [31, 32]). However, the way in which birds respond to noise pollution is not straightforward. An increasing number of studies indicate that the response can be case specific, and some species have been found to respond differently to high levels of natural versus anthropogenic noise, as well as different levels of the latter. For example, in the Pacific Wren *Troglodytes pacificus*, traffic noise had an effect on song duration but no effect on syllable length, while ocean noise influenced syllable length, but no effect was observed for song duration [33]. Common Chaffinch *Fringilla coelebs* increased their signal redundancy while singing near noisy mountain torrents [34], but not in noisy urban areas [35]. The song of Common Blackbirds *Turdus merula* living within inner city districts of Vienna differed in song spectral characteristics compared to those of birds singing in a forest outside the city [36]. However, no such change was observed for Common Blackbirds exposed to aircraft noise from Madrid airport, compared to the control population [37]. Similarly, Common Chiffchaffs *Phylloscopus collybita* sing with higher frequency and a decreased number of syllables within a song along noisy highways, and through experimental exposure to noise were shown to be capable of a real–time song frequency shift in response to urban noise [38]. A more recent study found that Common Chiffchaffs singing near airports suffered from noise-induced hearing loss, which caused them to sing songs with lower vocal frequencies and a decreased song rate, than birds from a nearby control population [39].

Perception of noise in certain species may vary with its source, amplitude and features like duration, predictability or overlap with daily activities [40, 41]. Some species may possess broader environmental tolerance to noise pollution. The ‘noise filter hypothesis’ predicts that species using higher frequency vocalizations are more tolerant to noise pollution since their songs and calls are less masked by low-frequency urban noise [42]. Francis [43] analysed data from 183 bird species from Europe, North America and the Caribbean and found that species communicating with low frequency vocalizations tended to avoid noisy areas, but species with higher frequency vocalizations responded less aggressively. A similar comparative study shows that North American passerines using a larger frequency bandwidth were more tolerant to noise pollution [44]. However, this does not mean that species with higher-pitched songs are more likely to be more abundant in areas with noise pollution [44–46]. Rather, it suggests that their song will be less vulnerable to noise masking, which may allow them to get the message across a noisy environment without the need to adjust their spectral and temporal song parameters.

**Fig 1:**
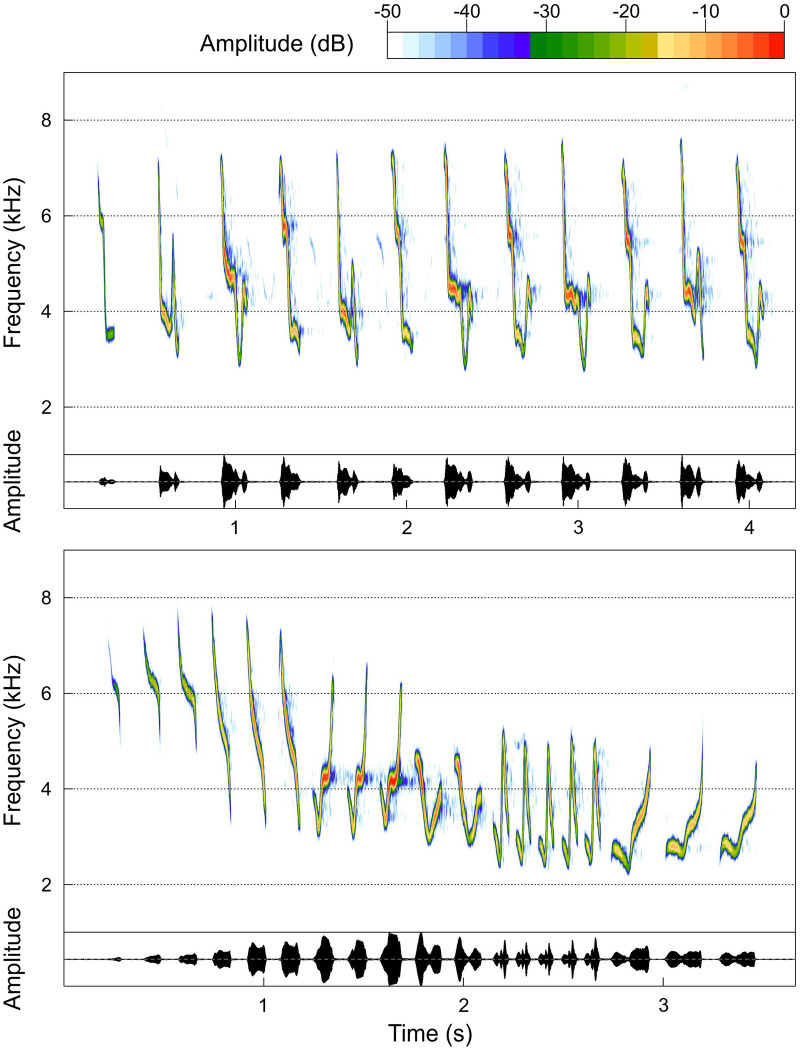
Spectrogram and oscillogram of typical Common Chiffchaff (upper) and Willow Warbler (lower) songs. Songs can differ from each other in the studied species, because they are composed of several different units, called syllables. The song of the Common Chiffchaff is composed of 12 syllables that belong to four unique syllable types. The song of the Willow Warbler is composed of 19 syllables that belong to six unique syllable types. Syllables can sometimes join into phrases, a repetition of a certain syllable type, as seen in the Willow Warbler song. Song nomenclature after Catchpole and Slater 2008 [20].

The main goal of our study was to evaluate the impact of moderate levels of noise pollution on the song of species which use vocalizations of higher frequency and large bandwidth. We think this is important, since high and severe levels of noise pollution are often limited to the close proximity of its source (i.e., roads, airports). However, the majority of urban green spaces which serve as suitable breeding areas for avian urban dwellers are usually polluted, with moderate noise levels reaching 50–55 dB (e.g., [47]). As model species we chose two closely related leaf warblers, the Common Chiffchaff *P*. *collybita* and the Willow Warbler *Phylloscopus trochilus*. We searched for differences in song characteristics between Common Chiffchaffs and Willow Warblers inhabiting urban green spaces affected by moderate noise pollution, and birds from nonurban natural forests. Territorial males of both species produce distinctive high frequency songs (Fig 1), and previous studies provide a decent background on their organisation and function (e.g., [38–53]). To date, noise-related song variation has been shown in two studies on the Common Chiffchaff, who modified their song frequency, song complexity and song rate under high (highway noise; [38]) and severe (airport noise; [39]) levels of noise pollution. These studies are crucial for two reasons. Firstly, we know that the Common Chiffchaff modifies its song in response to intense noise pollution. Secondly, various results indicate that the response to noise pollution in the Common Chiffchaff can be case specific. To our knowledge, the song of the Willow Warbler has not been previously tested in relation to noise pollution. Moreover, previous studies show that there are more issues in urban ecosystems that may affect acoustic communication than just noise, such as conspecific densities [54, 55], breeding status [55] or social factors in a given population [56]. Therefore we also described the frequency, repertoire and temporal organisation of the song output of studied species in relation to the day in the season, the time after sunrise and the presence of other vocally active males in the surroundings.

## Methods

### Study area

The study was conducted in the Wielkopolska Voivodeship in Western Poland. Urban populations were recorded in green spaces within the city of Poznań (N52.421617, E16.934186; S1 Fig). Urban sites consisted of park and woodland patches with a dominance of temperate and mixed coniferous forest, and were surrounded by a high density of urban development (housing, industry, major roads). Individuals were recorded up to 6 km from the city centre. Nonurban populations were localized in natural forests surrounding the city to the north, which consisted of two large mixed coniferous forests surrounded by farmland and rural areas: Zielonka Landscape Park (N52.562548, E17.120782; S2 Fig) and Notecka Forest Landscape Park (N52.727502, E16.721587; S3 Fig). Here, we avoided recording in areas near human settlements, roads or the recent or ongoing logging conducted by the State Forests. The distance from urban green spaces to nonurban forest study sites was between 15 and 40 km. Therefore, it was likely that potential differences in birdsong would originate from individual variation (e.g., social environment, quality) or micro-scale differences (e.g., noise, micro-habitat), rather than macro-scale differences (individuals belonging to populations with different dialects). All sites were open to the public and no permissions were required to access them during fieldwork.

### Song recording

Common Chiffchaffs were recorded between 2012–2015 from 11^th^ of April until 15^th^ of June, while Willow Warblers were recorded between 2013–2015 from 15^th^ of April until 15^th^ of June. When a singing male was localized it was approached to a distance of about 10–15 meters in order to record his song. Willow Warblers were usually stationary while singing, and sang from low trees or shrubs, Common Chiffchaff males sang from trees and in general were more mobile. If so, they were followed during the intervals between subsequent songs. There were no large differences in the times and dates of the recordings conducted in urban vs. forest habitats. Altogether, we recorded the songs of 61 Common Chiffchaffs (urban– 30, nonurban– 31) and 41 Willow Warblers (urban– 14, nonurban– 27). Recordings were conducted up to six hours after sunrise, on days with no rain and with low wind speeds (< 5 m/s, measured with a Voltcraft PL–130 anemometer; Conrad Electronics, Germany). Our study did not require approval by the Local Ethical Commission since recorded males were not captured and banded. In order to exclude the possibility of recording a particular bird more than once, sites where individuals were recorded were not visited again during the course of the study. Recordings were conducted with a Marantz PMD670 recorder (Marantz Professional, Japan) and a Telinga Pro 6 microphone mounted on a Telinga Universal parabola (Telinga Microphones, Sweden). Recordings were saved as mono–linear 48 kHz / 16 bit PCM WAV files.

### Song analysis

Spectrogram, oscillogram and amplitude scale displays were made with the R package “Seewave” [57]. During all acoustic analysis males were blind–coded so that the authors were unaware of their origin. We used Raven Pro 1.5 Beta v. 23 (Cornell Lab of Ornithology, USA) to conduct measurements of song duration (s), inter–song intervals (s), syllable durations (s) and inter–syllable intervals (s), as well as the number of syllables per song from 25 subsequent songs from each male of both studied species. We used the following spectrogram parameters to measure song characteristics: FFT length: 1024, window type: hamming, temporal overlap: 50%, time resolution: 10.7 ms, frequency resolution 46.9 Hz. The conducted measurements allowed us to calculate the song rate (number of songs produced per minute) and syllable rate (number of syllables produced per minute).

We measured the minimum frequency (Hz) and peak frequency (Hz) of all the syllables within 25 subsequent songs from each male. Measurements were conducted using the automatic parameter measurements function in Avisoft SASlab Pro v. 5.2.12 (Avisoft Bioacoustics, Germany) with the following spectrogram parameters: FFT length: 1024, frame size: 100%, window type: hamming, temporal overlap: 75%, time resolution: 5.33 ms, frequency resolution 46.9 Hz. A 1200 Hz high–pass filter was applied, and the amplitude threshold was set to –12 dB below the peak in a power spectrum. The value of the amplitude threshold was accepted after preliminary analysis of recordings with the lowest versus highest signal–to–noise ratio. Afterwards, we visually inspected the dataset to detect errors (i.e., incorrect measurements resulting from overlap with background noises).

Repertoire size was determined as the number of different syllables within the sample of 25 subsequent songs in both species. We used repertoire classification methodology from previous studies (e.g., [58, 59]). Syllables were classified on the basis of visual inspection of the spectrogram generated with Raven Pro (spectrogram parameters shown above). We then used the ratio of unique syllables to the total number of syllables per song to measure the song versatility index [60].

The songs of the Common Chiffchaff and Willow Warbler are delivered with a characteristic repetition pattern. We used the ratio of all transitions between unique syllable types to the sum of unique syllable types –1 per song to measure the redundancy index [28]. The redundancy index equals 1.0 when a bird continuously sings the same syllable type and equals 0 if a bird switches constantly between different syllable types. A linearity index was calculated taking the ratio of the number of unique syllable types to the number of transitions between different syllable types +1 per song [61]. A linearity index equals 1.0 when the syllable sequence is identical and reaches 0 when the syllable sequence is random.

### Noise analysis

The level of ambient noise present within each male’s territory was characterized immediately after the end of a song recording. Ten noise measurements were conducted after recording with a CHY 650 digital sound level meter (range: 35–130 dB SPL re 20 μPa; frequency weighting: A; fast response; ANSI S1.4, Class II), which were averaged for statistical analysis. Additionally, we compared ambient noise levels present in recorded males’ territories from urban and nonurban environments with Kaleidoscope Pro 5 (Wildlife Acoustics, Inc.) noise analysis functions. We measured minimum, mean and maximum sound pressure levels for 30 one-third octave bands between 19.7 Hz and 16000 Hz (see Kaleidoscope Pro manual for details) for 1 minute sampling periods. Each sampling period was prepared using recordings from each recorded male from both species, where the recorded male was silent and without any other species recorded as foreground. Thus all samples used in this analysis were recognized as recordings of ambient noise. Average values from each point were used for statistical analysis and figures were used to illustrate the differences in noise between urban and nonurban environments.

### Statistical analysis

To investigate the associations between selected predictors and song characteristics of both leaf warbler species, we performed generalized linear models (GLM) with the ‘stats’ package in R [62]. GLMs were run separately for each song characteristic and each species. We analysed the mean values of each song characteristic per male, and checked for multicollinearity problems by calculating variance inflation factors (VIF) on each full model. Environment type (urban/nonurban) and level of ambient noise showed multicollinearity (VIF value > 2). Therefore, we decided to exclude the environment type predictor and leave the ambient noise level in the further analysis. The predictor variables and covariates used in the GLMs were as follows: level of ambient noise present in the recorded male’s territory (average of ten noise level measurements conducted after recording), presence of other singing males within the hearing range (if the recorded male was singing alone = 0; singing with other nearby males = 1), day in a season and hour after sunrise.

A multi-model inference was used to identify the models which best described bird song variation. For this purpose Akaike’s information criterion corrected for small sample sizes (AIC_C_) and Akaike’s weight (*w*_*i*_) were calculated with the R package ‘MuMIn’ [63]. We considered models with Δ AIC_C_ less than 4 [64, 65], on which model averaging was performed. Model averaging is recommended when the weight of the best model is lower than 0.9 [66], which was true for our results. R-squared was also calculated for all models with the R package ‘rsq’ [67]. All other statistical analyses were performed using IBM SPSS Statistics v. 24 (IBM Corp, Chicago, IL, USA).

## Results

### Ambient noise in studied populations

Continuous noise at the urban site was associated with high-traffic on major city roadways. In addition, intermittent bursts of noise were caused by railroad transport, low-altitude airplanes using the local airport, car horns, car brakes, engines, sirens, wind gusts and bird song. Occasionally, passing trains covered entire frequency ranges on the recordings for a duration of up to 10 seconds. Continuous noise present in the nonurban population was connected with wind, while intermittent sounds were generated mainly by bird song and wind gusts, and occasionally by logging, high-altitude airplanes and low-altitude light aircraft. The mean level of ambient noise levels measured with a sound level meter differed between urban (50.6 ± 3.78 dB SPL, n = 30) and nonurban (39.8 ± 1.85 dB SPL, n = 31) Common Chiffchaff territories (Mann–Whitney U Test: Z = –6.680, P < 0.001), as well as urban (52.2 ± 4.48 dB SPL, n = 14) and nonurban (40.2 ± 1.79 dB SPL, n = 27) Willow Warbler territories (Mann–Whitney U Test: Z = –5.196, P < 0.001).

**Fig 2:**
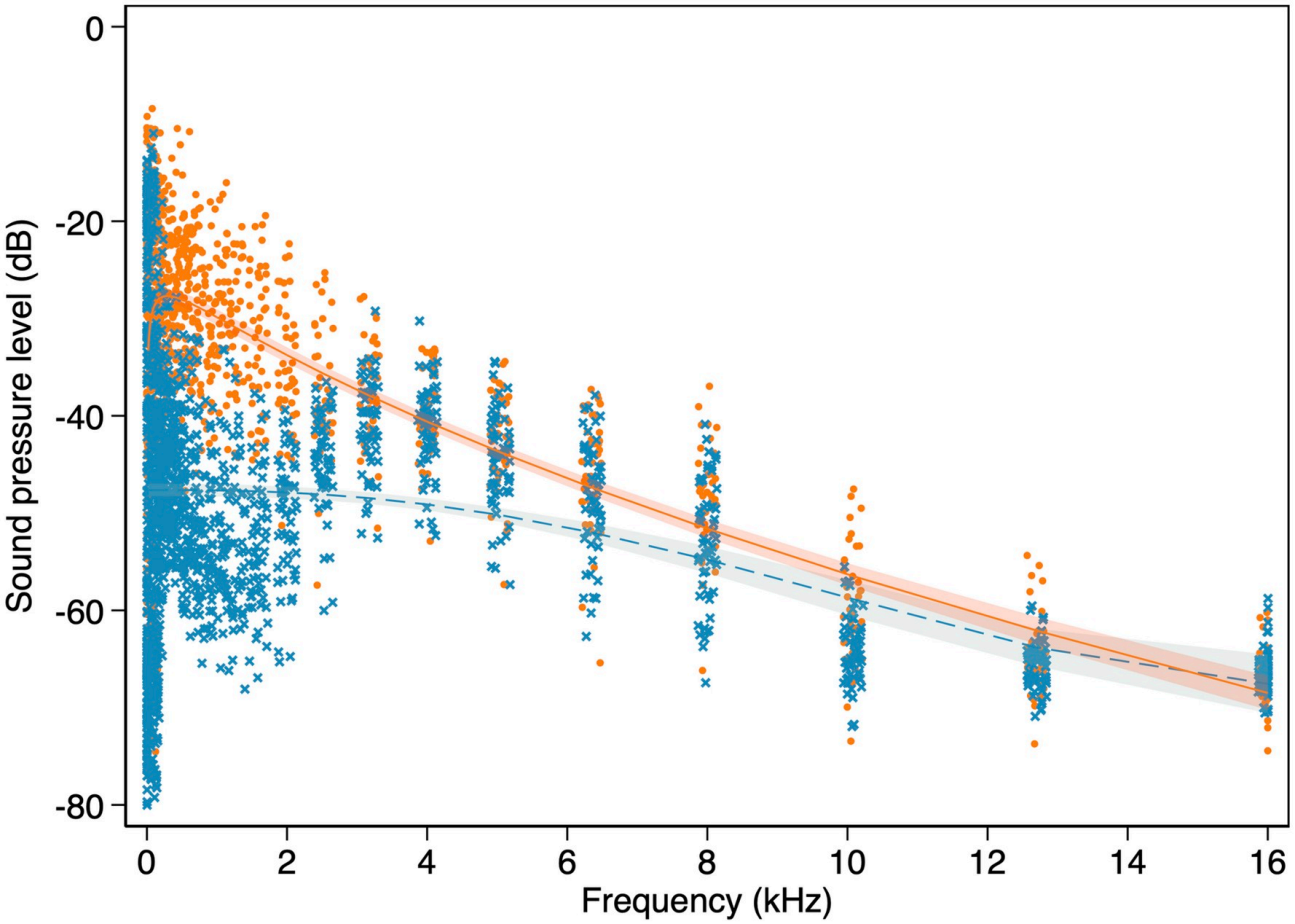
Comparison of mean sound pressure level (dB) measured for 30 one-third octave bands between 19.7 and 16000.0 kHz. Regression lines represent fractional polynomial fit with 95% confidence intervals. Urban data–orange dots and solid line; nonurban data–blue crosses and dashed line.

**Fig 3:**
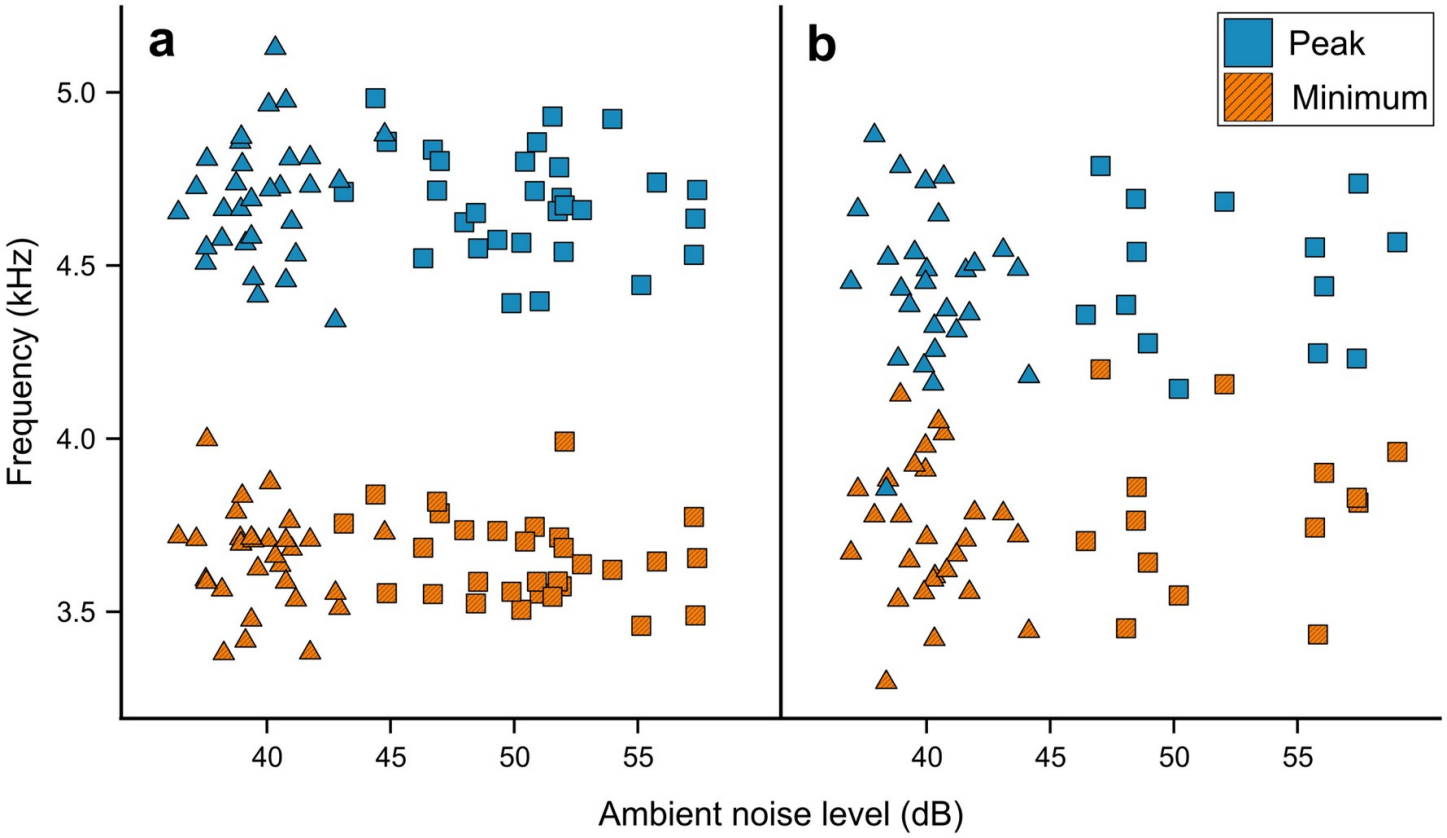
Relationship between the ambient noise level of Common Chiffchaffs (a) and Willow Warblers (b) syllable minimum and peak frequency.

**Fig 4:**
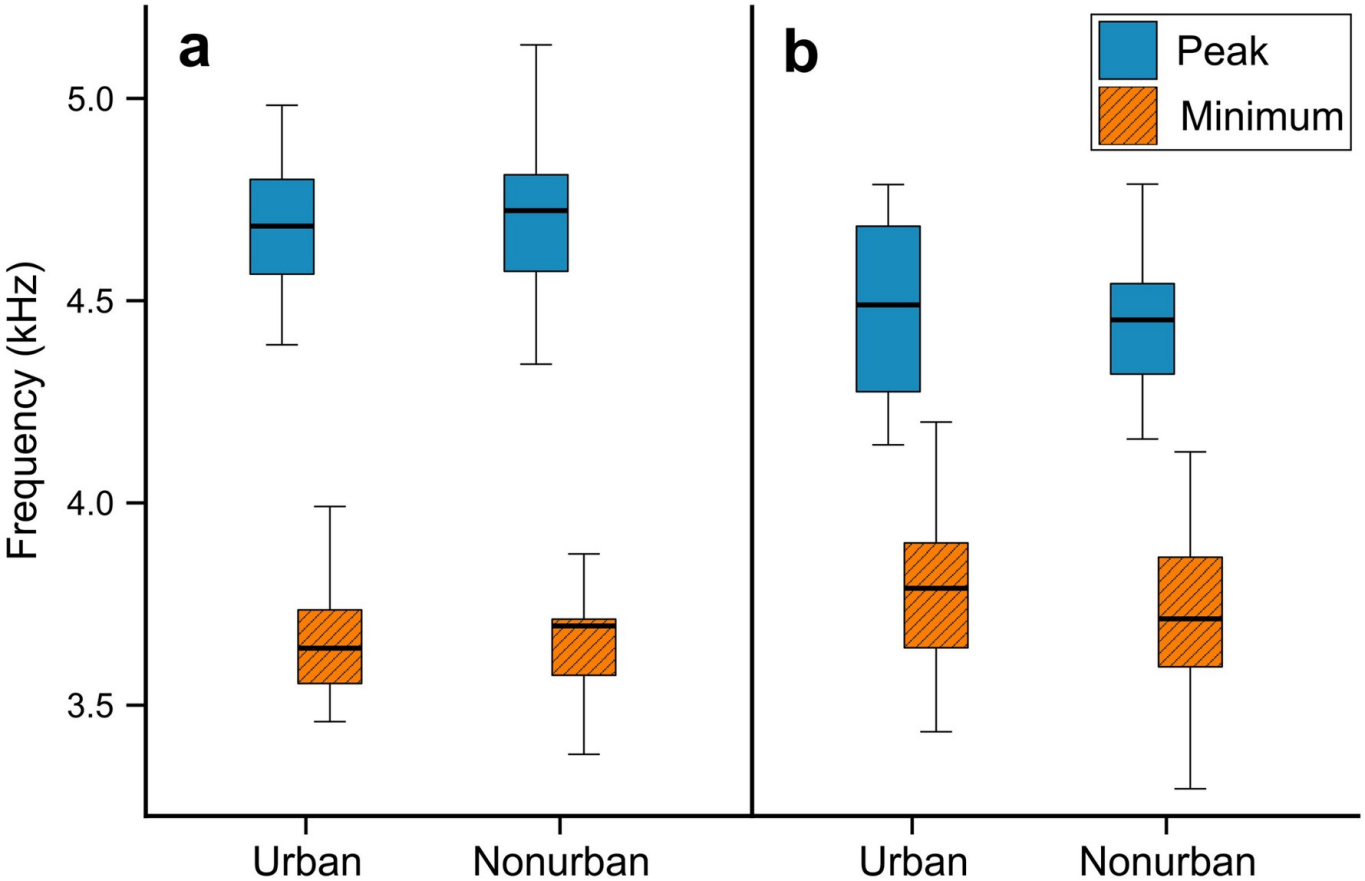
Box-and-whisker plots describing variation in Common Chiffchaffs (a) and Willow Warblers (b) syllable minimum and peak frequency in relation to sampling site. Boxes indicate median and first and third quartiles. Whiskers represent the minimal and maximal values within 1.5 times the interquartile range.

Noise analysis found that minimum, mean and maximum noise measured for 30 one-third octave bands were higher in urban (n = 44) than in nonurban (n = 58) sites in all bands from 19.7 Hz to 12699.2 Hz (Fig 2 and Table 1). The difference in ambient noise levels overlapping with the mean song frequency range of both the studied species reached only 2–3 dB (octave bands: 23–26; Table 1). The biggest differences (>20 dB) in noise levels between the studied sites were observed for frequencies between 49.6–78.7 Hz (octave bands: 5–7) and 500.0–1587.4 Hz (octave bands: 15–20; Table 1), which was below the studied species syllable minimum frequencies (Figs 3 and 4).

**Table 1:** Differences in average ambient noise levels between urban (n = 44) and nonurban (n = 58) recording sites.

Band no	Band Hz	Urban (dB)	Nonurban (dB)	Difference (dB)	F	P
1	19.7	-33.44	-48.21	14.77	8.59	0.004
2	24.8	-35.92	-50.91	14.99	8.16	0.005
3	31.2	-33.86	-48.56	14.70	11.57	0.001
4	39.4	-31.98	-49.51	17.53	61.41	<0.001
5	49.6	-26.33	-48.45	22.12	61.41	<0.001
6	65.5	-23.82	-48.51	24.69	96.86	<0.001
7	78.7	-26.01	-48.65	22.64	100.14	<0.001
8	99.2	-28.95	-47.98	19.03	88.54	<0.001
9	125.0	-30.10	-47.76	17.66	100.06	<0.001
10	157.3	-31.11	-47.16	16.05	111.38	<0.001
11	198.4	-30.39	-45.15	14.76	127.20	<0.001
12	250.0	-28.34	-45.73	17.39	159.82	<0.001
13	315.0	-26.65	-43.76	17.11	155.73	<0.001
14	396.9	-26.25	-44.30	18.05	187.99	<0.001
15	500.0	-27.88	-47.95	20.07	256.63	<0.001
16	630.0	-29.62	-51.02	21.40	225.35	<0.001
17	793.7	-30.08	-52.36	22.28	241.90	<0.001
18	1000.0	-28.99	-52.00	23.01	271.40	<0.001
19	1259.9	-30.58	-53.48	22.90	262.10	<0.001
20	1587.4	-32.19	-52.95	20.76	226.29	<0.001
21	2000.0	-34.93	-50.18	15.25	148.69	<0.001
22	2519.8	-37.73	-44.59	6.86	37.65	<0.001
23	3174.8	-38.19	-41.13	2.94	8.69	0.004
24	4000.0	-39.32	-41.46	2.14	5.44	0.022
25	5039.7	-42.32	-44.64	2.32	0.45	0.022
26	6349.6	-45.76	-48.43	2.67	5.54	0.021
27	8000.0	-48.50	-52.74	4.24	13.48	<0.001
28	10079.4	-60.06	-64.02	3.96	19.9	<0.001
29	12699.2	-64.44	-65.82	1.38	4.71	0.032
30	16000.0	-66.81	-66.07	-0.74	2.12	0.148

Ambient noise level was measured for 30 one-third octave bands for a 1 minute sampling period during which recorded leaf warblers were silent and without any other species recorded in the foreground. Octave bands from 23 to 26 overlapped with the mean song frequency range of both studied species.

### Differences in song characteristics in relation to ambient noise level

GLM analysis showed no relation between ambient noise level and the studied song characteristics of the Common Chiffchaff (S1 and S2 Tables) or the Willow Warbler (S3 and S4 Tables). The means and standard deviations of analysed song characteristics from studied populations are presented in S5 Table for the Common Chiffchaff, and in S6 Table for the Willow Warbler.

### Variation of the Common Chiffchaff song characteristics

The day in the season was the most important predictor, with 91% relative importance for the Common Chiffchaffs syllable minimum frequency (Table 2). This indicates that the mean syllable minimum frequency increased as the season advanced. The presence of males in the background had a 70% relative importance for syllable peak frequency, while day in the season had a 71% relative importance for syllable repertoire size. However, confidence intervals for the parameter estimates included zero in both cases, leaving little evidence that they affected these song characteristics. The relative importance of predictors in other song characteristics remained low, indicating no effect on the studied song characteristics (S1 and S2 Tables). General variation of Common Chiffchaff song characteristics is shown in S7 Table.

**Table 2:** Model-averaged estimates of factors describing variation in the Common Chiffchaff’s minimum syllable frequency.

Parameter	Estimate	SE	Confidence interval	Relative importance	N containing models
Intercept	3612.3814	60.6954	(3493.421, 3731.342)		
DAY	1.9938	0.8937	(0.242, 3.745)	0.91	4
MALES	-28.4290	51.3595	(-129.092, 72.234)	0.17	1
NOISE	-0.9879	2.7001	(-6.280, 4.304)	0.15	1
HOUR	-0.7709	12.5714	(-25.410, 23.869)	0.14	1

Model averaging was conducted on models with Δ AIC_C_ < 4. Abbreviations: DAY, day of season; HOUR, hour after sunrise; NOISE, background noise level; MALES, other singing males in hearing range; NULL, null model.

### Variation of Willow Warbler song characteristics

**Fig 5:**
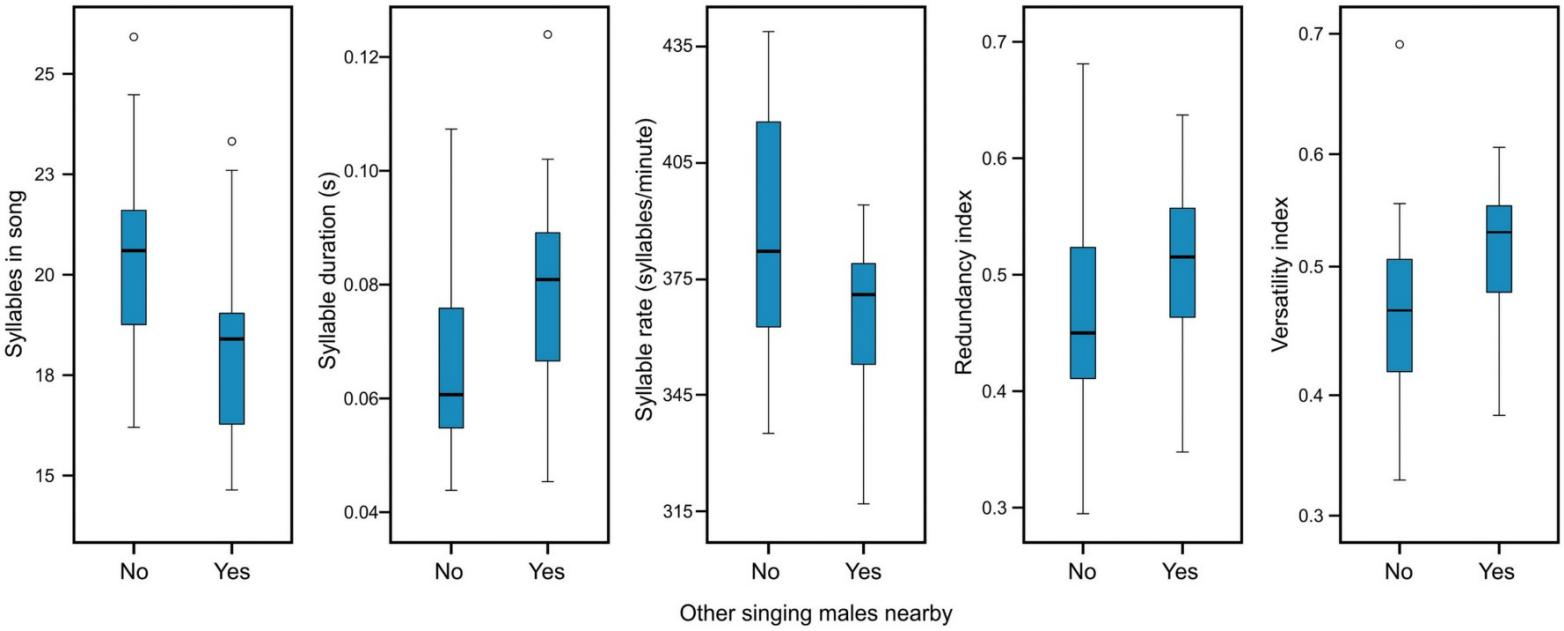
Box-and-whisker plots describing variation in Willow Warblers’ song characteristics in relation to the presence of other singing males nearby. Boxes indicate median and first and third quartiles. Whiskers represent the minimal and maximal values within 1.5 times the interquartile range. Open circles are outliers.

The presence of other singing males had a very high relative importance for the Willow Warblers syllable duration, syllable production rate and number of syllables within a song, as well as redundancy and versatility indices (Table 3). Songs of lone males (without a neighbour) were characterized by a higher number of syllables within a song, shorter syllable durations and higher syllable production rate (Fig 5). Lower values of the redundancy index in lone males corresponded with an increased switching between different syllable types, while lower values of versatility index indicated that lone males sang fewer unique syllable types per song (Fig 5). Furthermore, the hour after sunrise was the most important predictor for syllable peak frequency, which decreased as the day progressed (Table 3). While the hour after sunrise had a 73% and 70% relative importance for syllable minimum frequency and song duration, respectively, confidence intervals for the parameter estimates included zero in both cases. The relative importance of predictors in other song characteristics remained low, indicating no effect on the studied Willow Warblers song characteristics (S3 and S4 Tables). General variation of Willow Warbler song characteristics is shown in S8 Table.

**Table 3:** Model-averaged estimates of factors describing selected Willow Warbler song characteristics.

Parameter	Estimate	SE	Confidence interval	Relative importance	N containing models
PEAK FREQUENCY					
Intercept	4761.5693	178.7188	(4411.287, 5111.852)		
HOUR	-108.5736	42.2519	(-191.386, -25.762)	1.00	6
DAY	-2.5960	1.7660	(-6.058, 0.867)	0.49	3
NOISE	3.9930	5.2400	(-6.278, 14.264)	0.23	2
MALES	-28.3610	65.6490	(-157.030, 100.308)	0.18	2
SYLLABLES IN SONG					
Intercept	19.3878	1.7511	(15.956, 22.820)		
MALES	-2.1657	0.7933	(-3.721, -0.611)	1.00	6
DAY	0.0345	0.0213	(-0.007, 0.076)	0.55	3
HOUR	0.4729	0.5086	(-0.524, 1.470)	0.27	2
NOISE	-0.0002	0.0651	(-0.128, 0.128)	0.16	2
SYLLABLE DURATION					
Intercept	0.0578	0.0178	(0.023, 0.093)		
MALES	0.0129	0.0059	(0.002, 0.024)	0.88	6
HOUR	0.0071	0.0038	(-0.001, 0.014)	0.71	5
NOISE	-0.0004	0.0005	(-0.001, 0.001)	0.25	3
DAY	-0.0001	0.0002	(-0.001, 0.001)	0.18	2
SYLLABLE RATE					
Intercept	393.7100	20.2186	(354.082, 433.338)		
MALES	-17.7380	8.1151	(-33.643, -1.833)	0.86	6
HOUR	-7.3349	5.2080	(-17.542, 2.873)	0.47	4
NOISE	-0.2797	0.6614	(-1.576, 1.017)	0.17	2
DAY	0.0643	0.2210	(-0.369, 0.498)	0.16	2
REDUNDANCY INDEX					
Intercept	0.4236	0.0573	(0.311, 0.536)		
MALES	0.0524	0.0257	(0.002, 0.103)	0.83	6
DAY	0.0013	0.0007	(-0.001, 0.003)	0.68	4
HOUR	0.0136	0.0165	(-0.019, 0.046)	0.20	2
NOISE	0.0010	0.0020	(-0.003, 0.005)	0.15	2
VERSATILITY INDEX					
Intercept	0.4544	0.0463	(0.364, 0.545)		
MALES	0.0510	0.0218	(0.007, 0.095)	0.88	6
DAY	0.0009	0.0006	(-0.001, 0.002)	0.49	4
HOUR	0.0079	0.0146	(-0.021, 0.036)	0.18	2
NOISE	0.0003	0.0018	(-0.003, 0.004)	0.16	2

Model averaging was conducted on models with Δ AIC_C_ < 4. Abbreviations: DAY, day of season; HOUR, hour after sunrise; NOISE, background noise level; MALES, other singing males in hearing range; NULL, null model. Significant values are in bold.

## Discussion

In this study we searched for song variation in relation to ambient noise levels in urban and nonurban populations of the Common Chiffchaff and the Willow Warbler. Although ambient noise levels were higher in the urban environment, the difference in noise levels overlapping with the mean song frequency range of both leaf warblers was marginal between urban and nonurban study sites. We found no evidence for noise-related bird song divergence in the studied leaf warblers. Similar negative results to those presented in the current study have been shown for a few other songbird species (e.g., [68, 69]). Although in many cases such results remain in the shadow of the positive ones, they all contribute to a better understanding of animal communication in the urban world.

While we observed significantly higher noise levels in the urban environment, the difference in ambient noise level in the frequencies corresponding with the song frequency range of the studied species was minimal (2–3 dB SPL). A similar difference within song frequency range was observed in the previous study, where Common Chiffchaffs modified their song characteristics (about 3 dB at 4.0 kHz octave band), however the overall noise levels were higher, reaching about 58 dB [38]. Lack of noise-related bird song divergence may be due to the relatively low variation in amplitude, or other noise features present within the song frequency range of studied species. However, certain species like the Black-capped Chickadees *Poecile atricapillus*, were found to demonstrate a high degree of spectral and temporal call flexibility in response to expeimental traffic noise reaching about 50 dB [70]. Birds may also possess a noise level threshold beyond which they start to modify their song characteristics in a certain way. Such behaviour was observed in King Penguins *Aptenodytes patagonicus*, which live in harsh sub-Antarctic environments. Here, winds blow strongly throughout the year, generating a high level of background noise, and individuals were observed to increase the number of calls emitted and the number of syllables per call when the wind speed was higher than 7 meters per second [71]. However, King Penguins belong to the group of nonpasserines that are not vocal learners and their vocalizations are much less plastic than those of the passerine birds. An important model species of the latter are Zebra Finches *Taenopygia guttata*, and an experimental study conducted on individuals housed in acoustic chambers varying in noise level showed that exposure beyond a certain level of traffic noise negatively affected the song-learning brain regions Area X and HVC, as well as the tutor and tutee sequence of sound similarity [72]. However, while the song learning in Zebra Finches was impacted by noise, the effect of noise exposure on their song frequency was small. Future studies should focus on describing noise features and determining whether there is a difference in bird song variation in response to a certain level of noise pollution within and outside the song frequency range of the studied species.

Our data showed that on average Common Chiffchaffs’ syllable minimum frequency increased as the season advanced. In the Common Chiffchaff, fighting ability is demonstrated by using songs characterized by a lower peak frequency [53]. Therefore, more intense interactions between conspecifics could motivate males to produce songs with a lower peak frequency. An observed increase in minimum frequency through the season may therefore be related to reduced motivation and willingness to fight as the breeding season progresses. Variation in syllable minimum frequency may also be related to changes in the acoustic properties of habitat caused by vegetation. Growth of leaf surface area on trees and shrubs results in reflection and diffraction of lower frequencies, which may influence sound transmission properties [73, 74].

In the Willow Warbler, syllable peak frequency decreased throughout the course of a day. Previous studies on the Willow Warblers found that males are able to decrease song pitch when challenged [75]. Song pitch also indicates body size, and Willow Warblers were shown to react with more aggression when presented with lower–pitched songs [76]. Since we found no relationship between noise levels and syllable frequency, it could be possible that interactions between conspecifics vary during the day, which may be reflected in song spectral characteristics.

Most of the observed Willow Warblers’ song variation was explained by the presence of other singing males nearby. The singing behaviour of the Willow Warbler was previously shown to depend on population density, where males from high density populations had higher song outputs, and their songs were longer and more variable [22]. Living in higher densities increases the chance for interactions between individuals and fights over limited resources, which may be reflected in their acoustic signalling [77]. Male density was shown to affect the minimum frequency and the number of phrases in Great Tits *Parus major* [78]. Wood Thrushes *Hylocichla mustelina* and Ovenbirds *Seiurus aurocapilla* sang more often in high density populations [79]. However, the opposite was observed in the Corn Bunting *Emberiza calandra*, where higher male densities induced more interactions between neighbours, and instead of singing, males were actively deterring rivals from their territory with calls [80]. Similar results were shown for the Orange-Crowned Warbler *Leiothlypis celata*, where population density not only affected singing behaviour, but also territorial aggression in response to simulated territory intrusions [81]. Orange-Crowned Warblers living in high densities responded more aggressively towards the songs of neighbours than strangers, while birds from low density populations did the opposite. A positive relationship between population density and aggressiveness was observed in several species, like the Eurasian Oystercatchers *Haematopus ostralegus* [82], however in some cases this relationship did not appear [77]. Nevertheless, since song is an aggressive signal in songbirds [83] we should expect that it may reflect population density.

Although we found no evidence for noise-related bird song divergence, we show that social factors, and time in the day and during the season, influence certain Common Chiffchaff and Willow Warbler song characteristics. Our findings may be an outcome of the relatively low noise levels present at the urban study site, which did not provide sufficient noise masking of the song of the studied species. On the other hand, both leaf warblers may possess broader environmental tolerance to noise pollution due to the higher vocal frequencies of their songs. Future studies of urban bird song should focus on areas with more intense anthropogenic noise levels, which greatly overlap with the song frequency range. Further analysis should also include factors related to time in the day and during the season, as well as a description of population size and structure, which may shape the singing behaviour of the studied population. Such a relationship should not be undermined since it could lead to false positives in the study of bird song in urban ecosystems.

## Supporting information

S1 Table Results of generalised linear models assessing variation in Common Chiffchaff song characteristics. (DOCX)

S2 Table Model-averaged estimates of factors describing variation in Common Chiffchaff song characteristics. (DOCX)

S3 Table Results of generalised linear models assessing variation in Willow Warbler song characteristics. (DOCX)

S4 Table Model-averaged estimates of factors describing variation in Willow Warbler song characteristics. (DOCX)

S5 Table Song characteristics of Common Chiffchaff from urban (N = 30) and nonurban (N = 31) populations. (DOCX)

S6 Table Song characteristics of Willow Warbler from urban (N = 14) and nonurban (N = 27) populations. (DOCX)

S7 Table General variation in Common Chiffchaff song characteristics. (DOCX)

S8 Table General variation in Willow Warbler song characteristics. (DOCX)

S1 Fig Recording sites within the city of Poznań (N52.421617, E16.934186). (TIF)

S2 Fig Recording sites within the Zielonka Landscape Park (N52.562548, E17.120782). (TIF)

S3 Fig Recording sites within the Notecka Forest Landscape Park (N52.727502, E16.721587). (TIF)
