# Supplementary material for: Seasonality and social factors, but not noise pollution, influence the song characteristics of two leaf warbler species
Source: PLoS One. 2021 Sep 2;16(9):e0257074. doi: 10.1371/journal.pone.0257074 (PMC8412285; doi:10.1371/journal.pone.0257074)
Supplement: S1 Table — (DOCX) [file pone.0257074.s001.docx]

**S1 Table. Results of generalised linear models assessing variation in Common Chiffchaff song characteristics**

| **Predictors** | **Δ AIC_C_** | ***w_i_*** | ***R^2^*** |
| --- | --- | --- | --- |
| **PEAK FREQUENCY** |  |  |  |
| MALES | 0.00 | 0.32 | 0.061 |
| NULL | 1.65 | 0.14 |  |
| DAY + MALES | 1.98 | 0.12 | 0.066 |
| MALES + NOISE | 2.05 | 0.12 | 0.065 |
| HOUR + NOISE | 2.24 | 0.10 | 0.017 |
| NOISE | 3.43 | 0.06 | 0.007 |
| DAY | 3.66 | 0.05 | 0.003 |
| HOUR | 3.84 | 0.05 | <0.001 |
| HOUR + MALES + NOISE | 3.93 | 0.04 | 0.073 |
| **MINIMUM FREQUENCY** | | |  |
| DAY | 0.00 | 0.44 | 0.083 |
| DAY + MALES | 1.96 | 0.17 | 0.088 |
| DAY + NOISE | 2.15 | 0.15 | 0.086 |
| DAY + HOUR | 2.29 | 0.14 | 0.083 |
| NULL | 3.09 | 0.09 |  |
| **SONG DURATION** | | |  |
| NULL | 0.00 | 0.21 |  |
| DAY | 0.21 | 0.19 | 0.032 |
| MALES | 1.70 | 0.09 | 0.008 |
| DAY + HOUR | 1.84 | 0.08 | 0.043 |
| DAY + MALES | 1.87 | 0.08 | 0.042 |
| HOUR | 2.14 | 0.07 | 0.001 |
| NOISE | 2.14 | 0.07 | 0.001 |
| DAY + NOISE | 2.49 | 0.06 | 0.033 |
| DAY + HOUR + MALES | 3.51 | 0.04 | 0.054 |
| DAY + HOUR + NOISE | 3.62 | 0.03 | 0.052 |
| HOUR + MALES | 3.91 | 0.03 | 0.007 |
| MALES + NOISE | 3.95 | 0.03 | 0.009 |
| **SONG INTERVAL** | | |  |
| NULL | 0.00 | 0.18 |  |
| DAY | 0.67 | 0.13 | 0.025 |
| NOISE | 1.06 | 0.11 | 0.019 |
| DAY + HOUR + NOISE | 1.10 | 0.10 | 0.090 |
| HOUR + NOISE | 1.15 | 0.09 | 0.040 |
| HOUR | 2.09 | 0.06 | 0.002 |
| MALES | 2.10 | 0.06 | 0.002 |
| DAY + NOISE | 2.13 | 0.06 | 0.038 |
| DAY + HOUR | 2.28 | 0.06 | 0.036 |
| DAY + MALES | 2.88 | 0.04 | 0.026 |
| MALES + NOISE | 3.15 | 0.04 | 0.022 |
| DAY + HOUR + MALES + NOISE | 3.37 | 0.03 | 0.093 |
| HOUR + MALES + NOISE | 3.67 | 0.03 | 0.052 |
| **SONG RATE** | | |  |
| NULL | 0.00 | 0.36 |  |
| NOISE | 1.86 | 0.14 | 0.006 |
| MALES | 1.91 | 0.14 | 0.005 |
| HOUR | 2.18 | 0.12 | 0.001 |
| DAY | 2.21 | 0.12 | <0.001 |
| HOUR + MALES | 3.64 | 0.06 | 0.005 |
| MALES + NOISE | 3.78 | 0.05 | 0.012 |
| **SYLLABLES IN SONG** | | |  |
| NULL | 0.00 | 0.25 |  |
| DAY | 0.29 | 0.22 | 0.031 |
| DAY + HOUR | 1.92 | 0.10 | 0.042 |
| MALES | 2.04 | 0.09 | 0.003 |
| HOUR | 2.13 | 0.09 | 0.001 |
| NOISE | 2.19 | 0.08 | <0.001 |
| DAY + MALES | 2.34 | 0.08 | 0.035 |
| DAY + NOISE | 2.59 | 0.07 | 0.031 |
| DAY + HOUR + NOISE | 3.92 | 0.04 | 0.048 |
| **SYLLABLE DURATION** | | |  |
| NOISE | 0.00 | 0.22 | 0.038 |
| NULL | 0.12 | 0.20 |  |
| HOUR | 0.97 | 0.13 | 0.022 |
| MALES + NOISE | 2.18 | 0.07 | 0.039 |
| DAY + NOISE | 2.22 | 0.07 | 0.039 |
| HOUR + NOISE | 2.23 | 0.07 | 0.039 |
| MALES | 2.30 | 0.07 | <0.001 |
| DAY | 2.33 | 0.07 | 0.004 |
| DAY + HOUR | 3.04 | 0.05 | 0.026 |
| HOUR + MALES | 3.22 | 0.04 | 0.023 |
| **SYLLABLE INTERVAL** | | |  |
| MALES | 0.00 | 0.24 | 0.043 |
| NULL | 0.47 | 0.19 |  |
| DAY + MALES | 1.40 | 0.12 | 0.057 |
| DAY | 1.98 | 0.09 | 0.012 |
| HOUR + MALES | 2.29 | 0.08 | 0.043 |
| MALES + NOISE | 2.29 | 0.08 | 0.043 |
| NOISE | 2.66 | 0.06 | <0.001 |
| HOUR | 2.68 | 0.06 | <0.001 |
| DAY + HOUR + MALES | 3.70 | 0.04 | 0.058 |
| DAY + MALES + NOISE | 3.76 | 0.04 | 0.057 |
| **SYLLABLE RATE** | | |  |
| MALES | 0.00 | 0.14 | 0.041 |
| NULL | 0.34 | 0.12 |  |
| NOISE | 0.37 | 0.12 | 0.035 |
| MALES + NOISE | 0.44 | 0.11 | 0.070 |
| DAY + MALES | 0.96 | 0.09 | 0.062 |
| DAY | 1.44 | 0.07 | 0.018 |
| HOUR + MALES | 1.69 | 0.06 | 0.050 |
| DAY + MALES + NOISE | 1.87 | 0.06 | 0.084 |
| HOUR | 1.91 | 0.05 | 0.011 |
| DAY + NOISE | 1.91 | 0.05 | 0.047 |
| HOUR + MALES | 2.63 | 0.04 | 0.036 |
| HOUR + MALES + NOISE | 2.81 | 0.03 | 0.070 |
| DAY + HOUR + MALES | 3.17 | 0.03 | 0.064 |
| DAY + HOUR | 3.51 | 0.02 | 0.022 |
| **SYLLABLE REPERTOIRE SIZE** | | |  |
| DAY + MALES | 0.00 | 0.28 | 0.107 |
| DAY | 1.28 | 0.15 | 0.053 |
| MALES | 1.55 | 0.13 | 0.049 |
| DAY + HOUR + MALES | 2.02 | 0.10 | 0.112 |
| DAY + MALES + NOISE | 2.37 | 0.08 | 0.107 |
| NULL | 2.40 | 0.08 |  |
| DAY + HOUR | 3.34 | 0.05 | 0.057 |
| DAY + NOISE | 3.57 | 0.05 | 0.053 |
| MALES + NOISE | 3.81 | 0.04 | 0.049 |
| HOUR + MALES | 3.83 | 0.04 | 0.049 |
| **REDUNDANCY INDEX** | | |  |
| NULL | 0.00 | 0.23 |  |
| MALES | 0.40 | 0.19 | 0.029 |
| DAY | 1.74 | 0.10 | 0.008 |
| HOUR | 1.95 | 0.09 | 0.004 |
| DAY + MALES | 2.11 | 0.08 | 0.039 |
| NOISE | 2.20 | 0.08 | <0.001 |
| HOUR + MALES | 2.39 | 0.07 | 0.034 |
| MALES + NOISE | 2.70 | 0.06 | 0.029 |
| DAY + HOUR | 3.41 | 0.04 | 0.018 |
| DAY + HOUR + MALES | 3.73 | 0.04 | 0.050 |
| HOUR + NOISE | 3.91 | 0.03 | 0.010 |
| **LINEARITY INDEX** | | |  |
| NULL | 0.00 | 0.37 |  |
| HOUR | 1.77 | 0.15 | 0.007 |
| MALES | 2.09 | 0.13 | 0.002 |
| NOISE | 2.17 | 0.12 | <0.001 |
| DAY | 2.21 | 0.12 | <0.001 |
| HOUR + MALES | 3.95 | 0.05 | 0.009 |
| HOUR + NOISE | 3.98 | 0.05 | 0.009 |
| **VERSATILITY INDEX** | | |  |
| NULL | 0.00 | 0.34 |  |
| MALES | 1.55 | 0.16 | 0.011 |
| HOUR | 2.08 | 0.12 | 0.002 |
| NOISE | 2.10 | 0.12 | 0.002 |
| DAY | 2.14 | 0.12 | 0.001 |
| HOUR + MALES | 3.72 | 0.05 | 0.013 |
| DAY + MALES | 3.74 | 0.05 | 0.013 |
| MALES + NOISE | 3.77 | 0.05 | 0.012 |

Models with Δ AIC_C_ < 4 are shown. Abbreviations: AIC_c_, Akaike’s Information Criterion corrected for small sample size; w_i_, Akaike weight; DAY, day of season; HOUR, hour after sunrise; NOISE, background noise level; MALES, other singing males in hearing range during recording; NULL, null model.
