# Supplementary material for: Seasonality and social factors, but not noise pollution, influence the song characteristics of two leaf warbler species
Source: PLoS One. 2021 Sep 2;16(9):e0257074. doi: 10.1371/journal.pone.0257074 (PMC8412285; doi:10.1371/journal.pone.0257074)
Supplement: S2 Table — (DOCX) [file pone.0257074.s002.docx]

**S2 Table. Model-averaged estimates of factors describing variation in Common Chiffchaff song characteristics**

| **Parameter** | **Estimate** | **SE** | **Confidence interval** | **Relative importance** | **N containing models** |
| --- | --- | --- | --- | --- | --- |
| **PEAK FREQUENCY** | | | |  |  |
| Intercept | 4722.8280 | 102.3666 | (4522.193, 4923.463) |  |  |
| MALES | -126.6157 | 66.4437 | (-256.843, 3.613) | 0.70 | 5 |
| NOISE | -2.2017 | 3.8035 | (-9.656, 5.253) | 0.22 | 3 |
| HOUR | -5.3407 | 17.1407 | (-38.936, 28.255) | 0.20 | 3 |
| DAY | 0.5858 | 1.1566 | (-1.681, 2.853) | 0.17 | 2 |
| **MINIMUM FREQUENCY** | | | |  |  |
| Intercept | 3612.3814 | 60.6954 | (3493.421, 3731.342) |  |  |
| DAY | 1.9938 | 0.8937 | (0.242, 3.745) | 0.91 | 4 |
| MALES | -28.4290 | 51.3595 | (-129.092, 72.234) | 0.17 | 1 |
| NOISE | -0.9879 | 2.7001 | (-6.280, 4.304) | 0.15 | 1 |
| HOUR | -0.7709 | 12.5714 | (-25.410, 23.869) | 0.14 | 1 |
| **SONG DURATION** | | | | | |
| Intercept | 4.5315 | 0.8340 | (2.897, 6.166) |  |  |
| DAY | 0.0148 | 0.0104 | (-0.006, 0.035) | 0.49 | 6 |
| MALES | -0.4253 | 0.5883 | (-1.578, 0.728) | 0.27 | 5 |
| HOUR | -0.0939 | 0.1558 | (-0.399, 0.211) | 0.26 | 5 |
| NOISE | -0.0097 | 0.0338 | (-0.076, 0.057) | 0.20 | 4 |
| **SONG INTERVAL** | | | | | |
| Intercept | 4.7271 | 2.5502 | (-0.271, 9.725) |  |  |
| NOISE | 0.0653 | 0.0513 | (-0.035, 0.165) | 0.46 | 7 |
| DAY | -0.0176 | 0.0135 | (-0.044, 0.009) | 0.43 | 6 |
| HOUR | 0.2730 | 0.2494 | (-0.217, 0.761) | 0.38 | 6 |
| MALES | -0.2815 | 0.7527 | (-1.757, 1.194) | 0.21 | 5 |
| **SONG RATE** | | | | | |
| Intercept | 6.1888 | 0.8143 | (4.593, 7.785) |  |  |
| NOISE | -0.0181 | 0.0278 | (-0.072, 0.036) | 0.26 | 3 |
| MALES | 0.2656 | 0.4855 | (-0.686, 1.217) | 0.19 | 2 |
| HOUR | -0.0449 | 0.1292 | (-0.298, 0.208) | 0.18 | 2 |
| DAY | -0.0006 | 0.0084 | (-0.017, 0.016) | 0.12 | 1 |
| **SYLLABLES IN SONG** | | | | | |
| Intercept | 13.5840 | 2.2103 | (9.252, 17.916) |  |  |
| DAY | 0.0395 | 0.0285 | (-0.016, 0.096) | 0.49 | 5 |
| HOUR | -0.2580 | 0.4311 | (-1.103, 0.587) | 0.22 | 3 |
| NOISE | -0.0167 | 0.0932 | (-0.199, 0.166) | 0.19 | 3 |
| MALES | -0.7063 | 1.6239 | (-3.889, 2.476) | 0.17 | 2 |
| **SYLLABLE DURATION** | | | | | |
| Intercept | 0.1380 | 0.0118 | (0.115, 0.161) |  |  |
| NOISE | -0.0004 | 0.0003 | (-0.001, 0.001) | 0.43 | 4 |
| HOUR | 0.0010 | 0.0012 | (-0.001, 0.003) | 0.30 | 4 |
| DAY | -0.0001 | 0.0001 | (-0.001, 0.001) | 0.19 | 3 |
| MALES | 0.0011 | 0.0047 | (-0.008, 0.010) | 0.19 | 3 |
| **SYLLABLE INTERVAL** | | | | | |
| Intercept | 0.2135 | 0.0081 | (0.198, 0.229) |  |  |
| MALES | -0.0114 | 0.0071 | (-0.025, 0.003) | 0.56 | 6 |
| DAY | 0.0001 | 0.0001 | (-0.001, 0.001) | 0.29 | 4 |
| HOUR | -0.0001 | 0.0017 | (-0.003, 0.003) | 0.18 | 3 |
| NOISE | -0.0001 | 0.0004 | (-0.001, 0.001) | 0.18 | 3 |
| **SYLLABLE RATE** | | | | | |
| Intercept | 179.2285 | 8.8368 | (161.909, 196.548) |  |  |
| MALES | 5.4752 | 3.6089 | (-1.598, 12.549) | 0.52 | 7 |
| NOISE | 0.2546 | 0.2003 | (-0.138, 0.647) | 0.41 | 6 |
| DAY | -0.0611 | 0.0635 | (-0.186, 0.063) | 0.32 | 6 |
| HOUR | -0.3391 | 0.9883 | (-2.276, 1.598) | 0.24 | 6 |
| **SYLLABLE REPERTOIRE SIZE** | | | | | |
| Intercept | 6.5835 | 1.6081 | (3.432, 9.735) |  |  |
| DAY | 0.0425 | 0.0229 | (-0.002, 0.087) | 0.71 | 6 |
| MALES | -2.3306 | 1.3041 | (-4.887, 0.225) | 0.67 | 6 |
| HOUR | -0.1270 | 0.3270 | (-0.768, 0.514) | 0.19 | 3 |
| NOISE | -0.0004 | 0.0696 | (-0.137, 0.136) | 0.17 | 3 |
| **REDUNDANCY INDEX** | | | | | |
| Intercept | 0.8769 | 0.0567 | (0.766, 0.988) |  |  |
| MALES | -0.0578 | 0.0440 | (-0.144, 0.028) | 0.43 | 5 |
| HOUR | -0.0067 | 0.0110 | (-0.028, 0.015) | 0.27 | 5 |
| DAY | 0.0006 | 0.0008 | (-0.001, 0.002) | 0.25 | 4 |
| NOISE | -0.0004 | 0.0025 | (-0.005, 0.004) | 0.17 | 3 |
| **LINEARITY INDEX** | | | | | |
| Intercept | 0.4141 | 0.0715 | (0.274, 0.554) |  |  |
| HOUR | 0.0086 | 0.0134 | (-0.018, 0.035) | 0.25 | 3 |
| MALES | 0.0179 | 0.0545 | (-0.125, 0.089) | 0.18 | 2 |
| NOISE | -0.0001 | 0.0032 | (-0.006, 0.006) | 0.18 | 2 |
| DAY | -0.0001 | 0.0009 | (-0.002, 0.002) | 0.12 | 1 |
| **VERSATILITY INDEX** | | | | | |
| Intercept | 0.3662 | 0.0504 | (0.268, 0.465) |  |  |
| MALES | -0.0355 | 0.0453 | (-0.124, 0.053) | 0.31 | 4 |
| HOUR | 0.0036 | 0.0104 | (-0.017, 0.024) | 0.17 | 2 |
| NOISE | -0.0007 | 0.0024 | (-0.005, 0.004) | 0.17 | 2 |
| DAY | 0.0002 | 0.0009 | (-0.001, 0.002) | 0.17 | 2 |

Model averaging was conducted on models with Δ AIC_C_ < 4. Abbreviations: DAY, day of season; HOUR, hour after sunrise; NOISE, background noise level; MALES, other singing males in hearing range during recording; NULL, null model.
