# Supplementary material for: Seasonality and social factors, but not noise pollution, influence the song characteristics of two leaf warbler species
Source: PLoS One. 2021 Sep 2;16(9):e0257074. doi: 10.1371/journal.pone.0257074 (PMC8412285; doi:10.1371/journal.pone.0257074)
Supplement: S3 Table — (DOCX) [file pone.0257074.s003.docx]

**S3 Table. Results of generalised linear models assessing variation in Willow Warbler song characteristics**

| **Predictors** | **Δ AIC_C_** | ***w_i_*** | ***R^2^*** |
| --- | --- | --- | --- |
| **PEAK FREQUENCY** |  |  |  |
| HOUR | 0.00 | 0.30 | 0.150 |
| DAY + HOUR | 0.15 | 0.28 | 0.196 |
| DAY + HOUR + NOISE | 1.80 | 0.12 | 0.215 |
| HOUR + NOISE | 2.02 | 0.11 | 0.159 |
| HOUR + MALES | 2.28 | 0.10 | 0.153 |
| DAY + HOUR + MALES | 2.49 | 0.09 | 0.201 |
| **MINIMUM FREQUENCY** | | |  |
| HOUR | 0.00 | 0.28 | 0.090 |
| HOUR + NOISE | 1.03 | 0.17 | 0.122 |
| NULL | 1.55 | 0.13 |  |
| DAY + HOUR | 2.28 | 0.09 | 0.094 |
| HOUR + MALES | 2.46 | 0.08 | 0.091 |
| DAY + HOUR + NOISE | 3.20 | 0.06 | 0.131 |
| NOISE | 3.29 | 0.05 | 0.014 |
| HOUR + MALES + NOISE | 3.50 | 0.05 | 0.125 |
| DAY | 3.73 | 0.04 | 0.004 |
| MALES | 3.88 | 0.04 | <0.001 |
| **SONG DURATION** | | |  |
| DAY + HOUR + MALES | 0.00 | 0.20 | 0.226 |
| HOUR + MALES | 0.43 | 0.16 | 0.166 |
| DAY + HOUR | 1.29 | 0.10 | 0.149 |
| DAY + MALES | 1.45 | 0.10 | 0.145 |
| MALES | 1.72 | 0.08 | 0.087 |
| HOUR | 1.88 | 0.08 | 0.083 |
| DAY | 2.67 | 0.05 | 0.065 |
| DAY + HOUR + MALES + NOISE | 2.73 | 0.05 | 0.226 |
| HOUR + MALES + NOISE | 3.02 | 0.04 | 0.167 |
| NULL | 3.10 | 0.04 |  |
| DAY + HOUR + NOISE | 3.78 | 0.03 | 0.151 |
| HOUR + NOISE | 3.96 | 0.03 | 0.092 |
| MALES + NOISE | 3.96 | 0.03 | 0.091 |
| **SONG INTERVAL** | | |  |
| NULL | 0.00 | 0.23 |  |
| HOUR | 0.73 | 0.16 | 0.038 |
| MALES | 0.88 | 0.15 | 0.035 |
| HOUR + MALES | 1.74 | 0.09 | 0.072 |
| DAY | 2.10 | 0.08 | 0.006 |
| NOISE | 2.32 | 0.07 | <0.001 |
| DAY + HOUR | 2.95 | 0.05 | 0.044 |
| HOUR + NOISE | 3.08 | 0.05 | 0.041 |
| DAY + MALES | 3.15 | 0.05 | 0.039 |
| MALES + NOISE | 3.16 | 0.05 | 0.039 |
| HOUR + MALES + NOISE | 3.88 | 0.05 | 0.082 |
| **SONG RATE** | | |  |
| NULL | 0.00 | 0.18 |  |
| MALES | 0.32 | 0.15 | 0.048 |
| HOUR | 0.43 | 0.14 | 0.045 |
| HOUR + MALES | 0.86 | 0.12 | 0.092 |
| DAY | 1.74 | 0.07 | 0.014 |
| DAY + MALES | 2.26 | 0.06 | 0.060 |
| DAY + HOUR | 2.26 | 0.06 | 0.060 |
| NOISE | 2.27 | 0.06 | 0.002 |
| MALES + NOISE | 2.77 | 0.04 | 0.048 |
| HOUR + NOISE | 2.89 | 0.04 | 0.045 |
| DAY + HOUR + MALES | 2.90 | 0.04 | 0.104 |
| HOUR + MALES + NOISE | 3.32 | 0.03 | 0.095 |
| **SYLLABLES IN SONG** | | |  |
| DAY + MALES | 0.00 | 0.32 | 0.230 |
| MALES | 0.47 | 0.25 | 0.173 |
| DAY + HOUR + MALES | 1.56 | 0.15 | 0.249 |
| HOUR + MALES | 1.98 | 0.12 | 0.192 |
| DAY + MALES + NOISE | 2.58 | 0.09 | 0.230 |
| MALES + NOISE | 2.91 | 0.07 | 0.173 |
| **SYLLABLE DURATION** | | |  |
| HOUR + MALES | 0.00 | 0.34 | 0.197 |
| MALES | 1.38 | 0.17 | 0.118 |
| HOUR + MALES + NOISE | 1.77 | 0.14 | 0.213 |
| DAY + HOUR + MALES | 2.16 | 0.12 | 0.206 |
| HOUR | 3.33 | 0.06 | 0.075 |
| DAY + MALES | 3.43 | 0.06 | 0.127 |
| MALES + NOISE | 3.59 | 0.06 | 0.124 |
| HOUR + NOISE | 3.76 | 0.05 | 0.120 |
| **SYLLABLE INTERVAL** | | |  |
| DAY | 0.00 | 0.22 | 0.086 |
| DAY + MALES | 0.87 | 0.14 | 0.121 |
| DAY + HOUR | 1.31 | 0.11 | 0.111 |
| NULL | 1.35 | 0.11 |  |
| MALES | 2.03 | 0.08 | 0.040 |
| DAY + HOUR + MALES | 2.33 | 0.07 | 0.145 |
| DAY + NOISE | 2.43 | 0.06 | 0.087 |
| HOUR | 2.65 | 0.06 | 0.025 |
| DAY + MALES + NOISE | 3.21 | 0.04 | 0.126 |
| HOUR + MALES | 3.46 | 0.04 | 0.063 |
| NOISE | 3.66 | 0.03 | <0.001 |
| DAY + HOUR + NOISE | 3.77 | 0.03 | 0.114 |
| **SYLLABLE RATE** | | |  |
| MALES | 0.00 | 0.28 | 0.114 |
| HOUR + MALES | 0.14 | 0.26 | 0.163 |
| MALES + NOISE | 2.14 | 0.10 | 0.121 |
| DAY + MALES | 2.36 | 0.08 | 0.116 |
| NULL | 2.61 | 0.07 |  |
| DAY + HOUR + MALES | 2.64 | 0.07 | 0.050 |
| HOUR + MALES + NOISE | 2.64 | 0.07 | 0.165 |
| HOUR | 3.01 | 0.06 | 0.046 |
| **SYLLABLE REPERTOIRE** | | |  |
| NULL | 0.00 | 0.23 |  |
| HOUR | 0.18 | 0.21 | 0.051 |
| NOISE | 1.51 | 0.11 | 0.020 |
| DAY | 1.84 | 0.09 | 0.012 |
| DAY + HOUR | 2.11 | 0.08 | 0.063 |
| HOUR + NOISE | 2.19 | 0.08 | 0.062 |
| MALES | 2.26 | 0.07 | 0.002 |
| HOUR + MALES | 2.54 | 0.06 | 0.054 |
| DAY + NOISE | 3.65 | 0.04 | 0.028 |
| MALES + NOISE | 3.69 | 0.04 | 0.027 |
| **REDUNDANCY INDEX** | | |  |
| DAY + MALES | 0.00 | 0.33 | 0.180 |
| MALES | 1.68 | 0.14 | 0.092 |
| DAY + HOUR + MALES | 1.79 | 0.14 | 0.196 |
| DAY | 2.25 | 0.11 | 0.079 |
| DAY + MALES + NOISE | 2.46 | 0.10 | 0.192 |
| NULL | 3.31 | 0.06 |  |
| HOUR + MALES | 3.42 | 0.06 | 0.108 |
| MALES + NOISE | 3.68 | 0.05 | 0.103 |
| **LINEARITY INDEX** | | |  |
| NULL | 0.00 | 0.24 |  |
| DAY | 0.71 | 0.17 | 0.039 |
| NOISE | 1.15 | 0.14 | 0.028 |
| HOUR | 1.79 | 0.10 | 0.013 |
| MALES | 2.30 | 0.08 | <0.001 |
| DAY + NOISE | 2.37 | 0.07 | 0.057 |
| DAY + HOUR | 2.60 | 0.07 | 0.052 |
| DAY + MALES | 3.16 | 0.05 | 0.039 |
| HOUR + NOISE | 3.30 | 0.05 | 0.036 |
| MALES + NOISE | 3.61 | 0.04 | 0.029 |
| **VERSATILITY INDEX** | | |  |
| DAY + MALES | 0.00 | 0.27 | 0.171 |
| MALES | 0.00 | 0.27 | 0.119 |
| HOUR + MALES | 2.14 | 0.09 | 0.126 |
| DAY + HOUR + MALES | 2.25 | 0.09 | 0.178 |
| MALES + NOISE | 2.37 | 0.08 | 0.121 |
| DAY + MALES + NOISE | 2.60 | 0.07 | 0.171 |
| NULL | 2.87 | 0.06 |  |
| DAY | 3.33 | 0.05 | 0.045 |

Models with Δ AIC_C_ < 4 are shown. Abbreviations: AIC_c_, Akaike’s Information Criterion corrected for small sample size; w_i_, Akaike weight; DAY, day of season; HOUR, hour after sunrise; NOISE, background noise level; MALES, other singing males in hearing range during recording; NULL, null model.
