# Supplementary material for: Seasonality and social factors, but not noise pollution, influence the song characteristics of two leaf warbler species
Source: PLoS One. 2021 Sep 2;16(9):e0257074. doi: 10.1371/journal.pone.0257074 (PMC8412285; doi:10.1371/journal.pone.0257074)
Supplement: S4 Table — (DOCX) [file pone.0257074.s004.docx]

**S4 Table. Model-averaged estimates of factors describing variation in Willow Warbler song characteristics**

| **Parameter** | **Estimate** | **SE** | **Confidence interval** | **Relative importance** | **N containing models** |
| --- | --- | --- | --- | --- | --- |
| **PEAK FREQUENCY** | | | |  |  |
| Intercept | 4761.5693 | 178.7188 | (4411.287, 5111.852) |  |  |
| HOUR | -108.5736 | 42.2519 | (-191.386, -25.762) | 1.00 | 6 |
| DAY | -2.5960 | 1.7660 | (-6.058, 0.867) | 0.49 | 3 |
| NOISE | 3.9930 | 5.2400 | (-6.278, 14.264) | 0.23 | 2 |
| MALES | -28.3610 | 65.6490 | (-157.030, 100.308) | 0.18 | 2 |
| **MINIMUM FREQUENCY** | | | |  |  |
| Intercept | 3840.1109 | 211.6608 | (3425.263, 4254.959) |  |  |
| HOUR | -84.3795 | 43.0862 | (-168.827, 0.068) | 0.73 | 6 |
| NOISE | 5.6720 | 5.3273 | (-4.769, 16.113) | 0.33 | 4 |
| DAY | -0.8304 | 1.8377 | (-4.432, 2.772) | 0.19 | 3 |
| MALES | 9.2823 | 68.3395 | (-124.660, 143.225) | 0.17 | 3 |
| **SONG DURATION** | | | | | |
| Intercept | 2.7834 | 0.3465 | (2.105, 3.642) |  |  |
| HOUR | 0.1417 | 0.0768 | (-0.009, 0.292) | 0.70 | 8 |
| MALES | -0.2195 | 0.1192 | (-0.453, 0.014) | 0.66 | 7 |
| DAY | 0.0052 | 0.0032 | (-0.001, 0.012) | 0.53 | 6 |
| NOISE | 0.0019 | 0.0101 | (-0.018, 0.022) | 0.18 | 5 |
| **SONG INTERVAL** | | | | | |
| Intercept | 6.9281 | 0.9765 | (5.014, 8.842) |  |  |
| HOUR | 0.3251 | 0.2688 | (-0.202, 0.852) | 0.38 | 5 |
| MALES | -0.4836 | 0.4188 | (-1.304, 0.337) | 0.37 | 5 |
| NOISE | -0.0104 | 0.0339 | (-0.077, 0.056) | 0.20 | 4 |
| DAY | 0.0051 | 0.0114 | (0.017, 0.027) | 0.18 | 3 |
| **SONG RATE** | | | | | |
| Intercept | 6.3995 | 0.6509 | (5.124, 7.675) |  |  |
| MALES | 0.3679 | 0.2744 | (-0.170, 0.906) | 0.45 | 6 |
| HOUR | -0.2317 | 0.1766 | (-0.578, 0.115) | 0.44 | 6 |
| DAY | -0.0053 | 0.0075 | (-0.020, 0.009) | 0.23 | 4 |
| NOISE | 0.0003 | 0.0225 | (-0.044, 0.044) | 0.18 | 4 |
| **SYLLABLES IN SONG** | | | | | |
| Intercept | 19.3878 | 1.7511 | (15.956, 22.820) |  |  |
| MALES | -2.1657 | 0.7933 | (-3.721, -0.611) | 1.00 | 6 |
| DAY | 0.0345 | 0.0213 | (-0.007, 0.076) | 0.55 | 3 |
| HOUR | 0.4729 | 0.5086 | (-0.524, 1.470) | 0.27 | 2 |
| NOISE | -0.0002 | 0.0651 | (-0.128, 0.128) | 0.16 | 2 |
| **SYLLABLE DURATION** | | | | | |
| Intercept | 0.0578 | 0.0178 | (0.023, 0.093) |  |  |
| MALES | 0.0129 | 0.0059 | (0.002, 0.024) | 0.88 | 6 |
| HOUR | 0.0071 | 0.0038 | (-0.001, 0.014) | 0.71 | 5 |
| NOISE | -0.0004 | 0.0005 | (-0.001, 0.001) | 0.25 | 3 |
| DAY | -0.0001 | 0.0002 | (-0.001, 0.001) | 0.18 | 2 |
| **SYLLABLE INTERVAL** | | | | | |
| Intercept | 0.0672 | 0.0099 | (0.048, 0.087) |  |  |
| DAY | -0.0002 | 0.0001 | (-0.001, 1.400) | 0.68 | 7 |
| MALES | 0.0052 | 0.0043 | (-0.003, 0.014) | 0.37 | 5 |
| HOUR | -0.0028 | 0.0028 | (-0.008, 0.003) | 0.31 | 5 |
| NOISE | 0.0001 | 0.0004 | (-0.001, 0.001) | 0.18 | 4 |
| **SYLLABLE RATE** | | | | | |
| Intercept | 393.7100 | 20.2186 | (354.082, 433.338) |  |  |
| MALES | -17.7380 | 8.1151 | (-33.643, -1.833) | 0.86 | 6 |
| HOUR | -7.3349 | 5.2080 | (-17.542, 2.873) | 0.47 | 4 |
| NOISE | -0.2797 | 0.6614 | (-1.576, 1.017) | 0.17 | 2 |
| DAY | 0.0643 | 0.2210 | (-0.369, 0.498) | 0.16 | 2 |
| **SYLLABLE REPERTOIRE SIZE** | | | | | |
| Intercept | 36.2348 | 19.2127 | (-1.421, 76.891) |  |  |
| HOUR | 6.3996 | 4.6560 | (-2.726, 15.525) | 0.43 | 4 |
| NOISE | 0.4521 | 0.5762 | (-0.677, 1.582) | 0.26 | 4 |
| DAY | 0.1282 | 0.1984 | (-0.261, 0.517) | 0.21 | 3 |
| MALES | 2.3727 | 7.3849 | (-12.102, 16.847) | 0.17 | 3 |
| **REDUNDANCY INDEX** | | | | | |
| Intercept | 0.4236 | 0.0573 | (0.311, 0.536) |  |  |
| MALES | 0.0524 | 0.0257 | (0.002, 0.103) | 0.83 | 6 |
| DAY | 0.0013 | 0.0007 | (-0.001, 0.003) | 0.68 | 4 |
| HOUR | 0.0136 | 0.0165 | (-0.019, 0.046) | 0.20 | 2 |
| NOISE | 0.0010 | 0.0020 | (-0.003, 0.005) | 0.15 | 2 |
| **LINEARITY INDEX** | | | | | |
| Intercept | 0.9960 | 0.0413 | (0.915, 1.077) |  |  |
| DAY | -0.0005 | 0.0004 | (-0.001, 0.001) | 0.36 | 4 |
| NOISE | -0.0012 | 0.0012 | (-0.004, 0.001) | 0.30 | 4 |
| HOUR | -0.0067 | 0.0102 | (-0.027, 0.013) | 0.21 | 3 |
| MALES | 0.0016 | 0.0159 | (-0.030, 0.033) | 0.17 | 3 |
| **VERSATILITY INDEX** | | | | | |
| Intercept | 0.4544 | 0.0463 | (0.364, 0.545) |  |  |
| MALES | 0.0510 | 0.0218 | (0.007, 0.095) | 0.88 | 6 |
| DAY | 0.0009 | 0.0006 | (-0.001, 0.002) | 0.49 | 4 |
| HOUR | 0.0079 | 0.0146 | (-0.021, 0.036) | 0.18 | 2 |
| NOISE | 0.0003 | 0.0018 | (-0.003, 0.004) | 0.16 | 2 |

Model averaging was conducted on models with Δ AIC_C_ < 4. Abbreviations: DAY, day of season; HOUR, hour after sunrise; NOISE, background noise level; MALES, other singing males in hearing range during recording; NULL, null model.
