# Supplementary material for: Seasonality and social factors, but not noise pollution, influence the song characteristics of two leaf warbler species
Source: PLoS One. 2021 Sep 2;16(9):e0257074. doi: 10.1371/journal.pone.0257074 (PMC8412285; doi:10.1371/journal.pone.0257074)
Supplement: S5 Table — (DOCX) [file pone.0257074.s005.docx]

**S5 Table. Song characteristics of Common Chiffchaff from urban (N=30) and nonurban (N=31) populations**

| **Variable** | **Urban** | **Nonurban** |
| --- | --- | --- |
| Syllable minimum frequency (Hz) | 3652.9 ± 120.80 | 3652.6 ± 138.03 |
| Syllable peak frequency (Hz) | 4682.6 ± 153.54 | 4698.6 ± 177.28 |
| Song duration (s) | 4.5 ± 1.68 | 4.5 ± 1.18 |
| Inter–song intervals (s) | 6.5 ± 2.07 | 5.9 ± 2.30 |
| Song rate (songs/min) | 5.8 ± 1.27 | 6.1 ± 1.07 |
| Syllables in song | 13.9 ± 4.46 | 13.7 ± 3.44 |
| Syllable duration (s) | 0.13 ± 0.011 | 0.13 ± 0.011 |
| Inter–syllable intervals (s) | 0.21 ± 0.016 | 0.21 ± 0.019 |
| Syllable rate (syllables/min) | 185.1 ± 10.63 | 182.1 ± 6.62 |
| Repertoire size | 7.2 ± 3.77 | 7.0 ± 2.75 |
| Versatility index | 0.36 ± 0.109 | 0.37 ± 0.112 |
| Linearity index | 0.41 ± 0.133 | 0.43 ± 0.133 |
| Redundancy index | 0.87 ± 0.099 | 0.87 ± 0.117 |

Data shown as mean ± SD.
