# Supplementary material for: Seasonality and social factors, but not noise pollution, influence the song characteristics of two leaf warbler species
Source: PLoS One. 2021 Sep 2;16(9):e0257074. doi: 10.1371/journal.pone.0257074 (PMC8412285; doi:10.1371/journal.pone.0257074)
Supplement: S6 Table — (DOCX) [file pone.0257074.s006.docx]

**S6 Table. Song characteristics of Willow Warbler from urban (N=14) and nonurban (N=27) populations**

| **Variable** | **Urban** | **Nonurban** |
| --- | --- | --- |
| Syllable minimum frequency (Hz) | 3786.4 ± 229.28 | 3724.5 ± 201.47 |
| Syllable peak frequency (Hz) | 4474.2 ± 207.52 | 4445.9 ± 223.90 |
| Song duration (s) | 3.1 ± 0.36 | 3.1 ± 0.41 |
| Inter–song intervals (s) | 7.2 ± 1.31 | 7.1 ± 1.30 |
| Song rate (songs/min) | 6.1 ± 0.86 | 6.2 ± 0.87 |
| Syllables in song | 19.6 ± 2.43 | 19.2 ± 2.82 |
| Syllable duration (s) | 0.07 ± 0.018 | 0.08 ± 0.020 |
| Inter–syllable intervals (s) | 0.06 ± 0.012 | 0.06 ± 0.015 |
| Syllable rate (syllables/min) | 378.8 ± 25.38 | 373.4 ± 26.86 |
| Repertoire size | 54.6 ± 18.25 | 47.7 ± 24.29 |
| Versatility index | 0.48 ± 0.062 | 0.50 ± 0.079 |
| Linearity index | 0.95 ± 0.070 | 0.98 ± 0.029 |
| Redundancy index | 0.48 ± 0.067 | 0.49 ± 0.092 |

Data shown as mean ± SD.
