# Supplementary material for: Seasonality and social factors, but not noise pollution, influence the song characteristics of two leaf warbler species
Source: PLoS One. 2021 Sep 2;16(9):e0257074. doi: 10.1371/journal.pone.0257074 (PMC8412285; doi:10.1371/journal.pone.0257074)
Supplement: S7 Table — (DOCX) [file pone.0257074.s007.docx]

**S7 Table. General variation in Common Chiffchaffs song characteristics (N=61)**

| **Variable** | **Mean** | **SD** | **Min** | **Max** |
| --- | --- | --- | --- | --- |
| Syllable minimum frequency (Hz) | 3652.8 | 128.76 | 3378.6 | 3998.5 |
| Syllable peak frequency (Hz) | 4690.7 | 164.84 | 4343.0 | 5132.5 |
| Song duration (s) | 4.6 | 1.43 | 2.7 | 10.7 |
| Inter-song intervals (s) | 6.2 | 1.82 | 3.7 | 12.8 |
| Song rate (songs/min) | 6.0 | 1.18 | 3.7 | 8.9 |
| Syllables in song | 13.8 | 3.94 | 8.5 | 29.5 |
| Syllable duration (s) | 0.13 | 0.011 | 0.11 | 0.16 |
| Inter-syllable intervals (s) | 0.21 | 0.018 | 0.17 | 0.26 |
| Syllable rate (syllables/min) | 183.6 | 8.88 | 157.9 | 201.6 |
| Repertoire size | 7.1 | 3.26 | 3 | 23 |
| Versatility index | 0.36 | 0.110 | 0.19 | 0.62 |
| Linearity index | 0.42 | 0.132 | 0.19 | 0.72 |
| Redundancy index | 0.87 | 0.107 | 0.55 | 1.00 |
