# Supplementary material for: Seasonality and social factors, but not noise pollution, influence the song characteristics of two leaf warbler species
Source: PLoS One. 2021 Sep 2;16(9):e0257074. doi: 10.1371/journal.pone.0257074 (PMC8412285; doi:10.1371/journal.pone.0257074)
Supplement: S8 Table — (DOCX) [file pone.0257074.s008.docx]

**S8 Table. General variation in Willow Warblers song characteristics (N=41)**

| **Variable** | **Mean** | **SD** | **Min** | **Max** |
| --- | --- | --- | --- | --- |
| Syllable minimum frequency (Hz) | 3745.7 | 210.59 | 3293.3 | 4199.9 |
| Syllable peak frequency (Hz) | 4455.6 | 216.25 | 3853.2 | 4877.8 |
| Song duration (s) | 3.1 | 0.39 | 2.5 | 4.3 |
| Inter-song intervals (s) | 7.1 | 1.29 | 5.4 | 9.7 |
| Song rate (songs/min) | 6.2 | 0.85 | 4.4 | 7.6 |
| Syllables in song | 19.3 | 2.67 | 14.6 | 25.9 |
| Syllable duration (s) | 0.07 | 0.019 | 0.04 | 0.12 |
| Inter-syllable intervals (s) | 0.06 | 0.014 | 0.04 | 0.10 |
| Syllable rate (syllables/min) | 375.2 | 26.18 | 316.9 | 438.5 |
| Repertoire size | 50.1 | 22.41 | 17 | 99 |
| Versatility index | 0.50 | 0.073 | 0.33 | 0.69 |
| Linearity index | 0.97 | 0.048 | 0.73 | 1.00 |
| Redundancy index | 0.49 | 0.084 | 0.29 | 0.68 |
