# Supplementary figures and images for: Seasonality and social factors, but not noise pollution, influence the song characteristics of two leaf warbler species
Source: PLoS One. 2021 Sep 2;16(9):e0257074. doi: 10.1371/journal.pone.0257074 (PMC8412285; doi:10.1371/journal.pone.0257074)

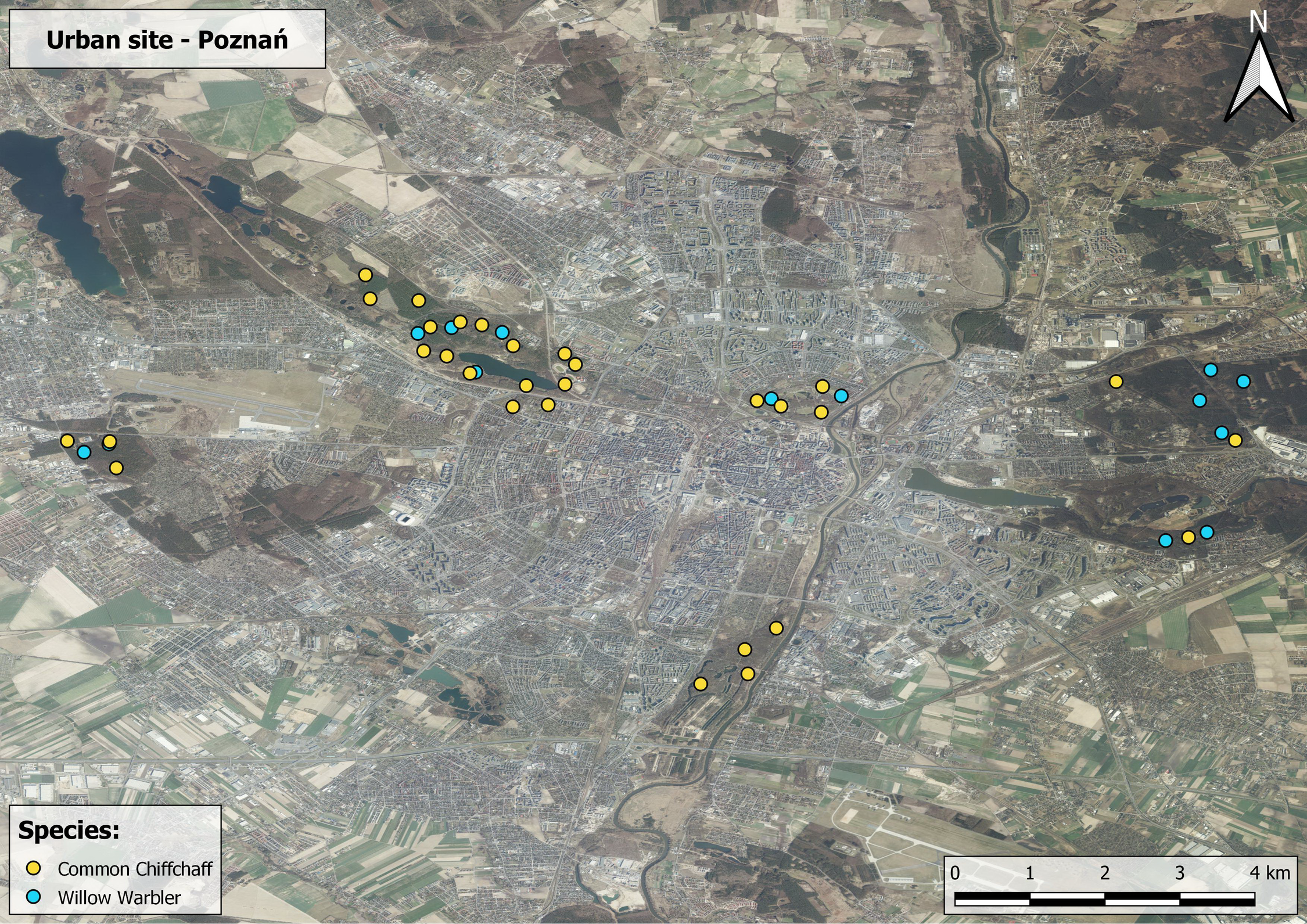

Supplement: S1 Fig — (TIF) [file pone.0257074.s009.tif]

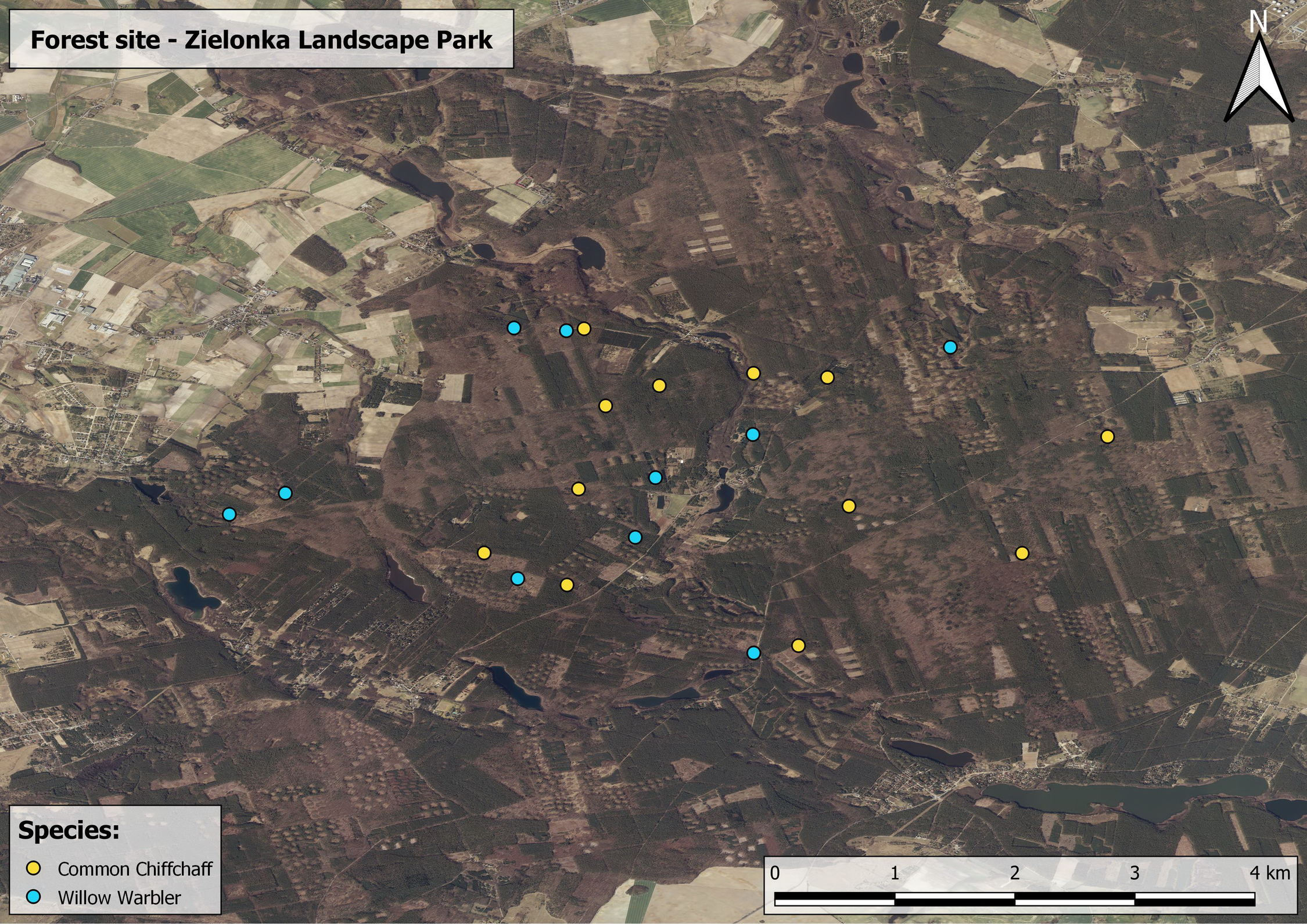

Supplement: S2 Fig — (TIF) [file pone.0257074.s010.tif]

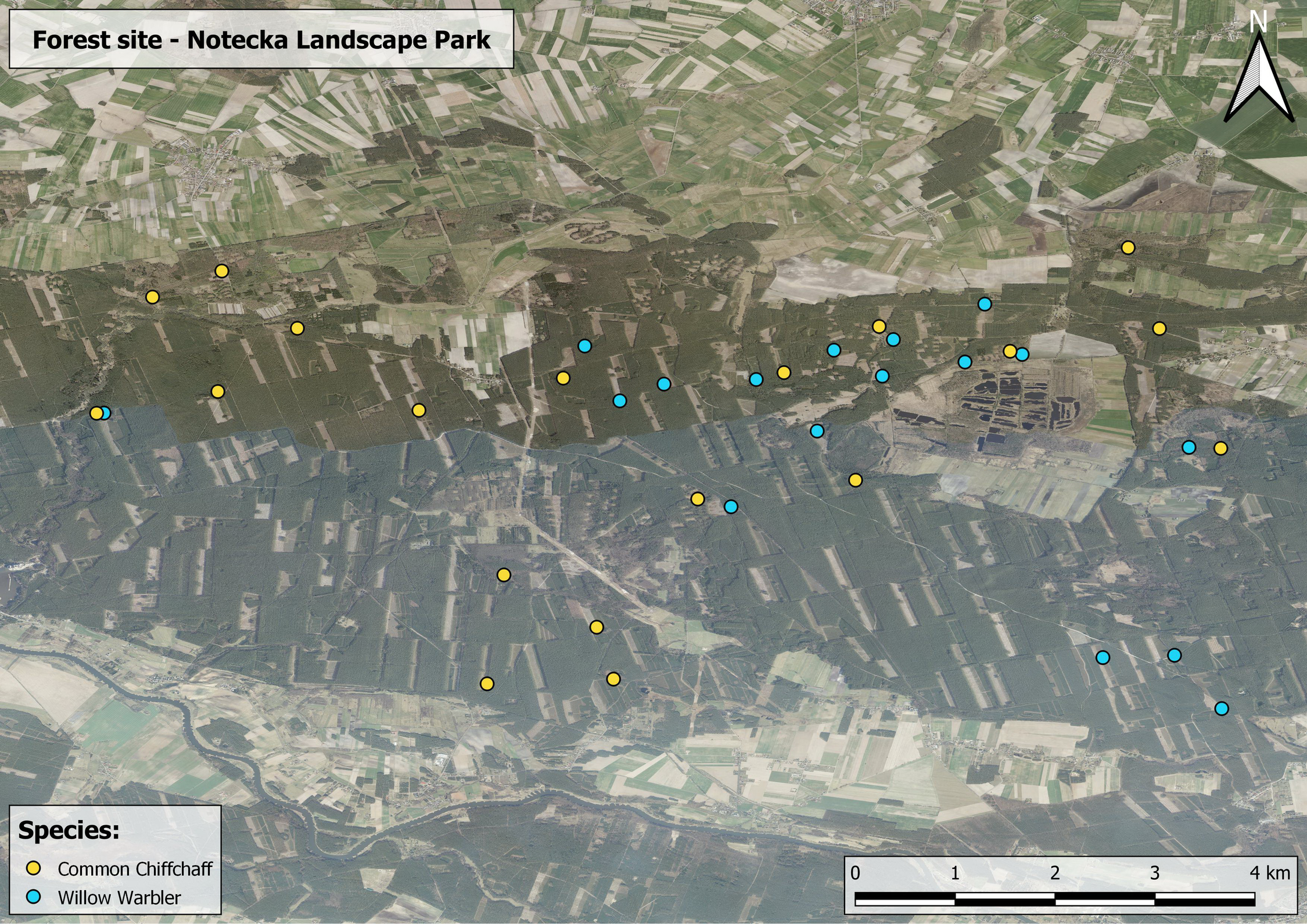

Supplement: S3 Fig — (TIF) [file pone.0257074.s011.tif]
